# The District Hospitals for the Insane in Ireland.—Superannuations

**Published:** 1856-10-01

**Authors:** 


### THE DISTRICT HOSPITALS FOR THE INSANE IN IRELAND. SUPERANNUATIONS.

In our last number we called particular attention to "No. 2 Bill," then before Parliament, "to explain and amend the Acts relating to Lunatic Asylums in Ireland."

That Bill, we have now to state, was withdrawn by Government, owing to the manner in which it had been mutilated in its original fair proportions after passing through Committee in the House of Commons. For several reasons we do not lament this result. One is, that we decidedly objected to the clause it contained of interfering so seriously with the existing admirable management of the Irish Asylums, by endeavouring to have introduced into them an element of discord by mixing together pay and pauper patients—a change which, we take for granted, could never have received the sanction or approval of the able and thoroughly practical Resident Physicians of those establishments, or of the Government Inspectors, Doctors White and Nugent. A plan of procedure more clumsy or utterly destructive to all harmony of action could not have been conceived, or one worse calculated to be a relief to the parties for whom it was intended to serve—viz., patients neither paupers nor in independent circumstances. At another time we may more fully go into this point, contenting ourselves for the present in again simply protesting against what was proposed to Parliament in this respect. A second reason we have for rejoicing rather at "No. 2" Bill sharing the fate of the "Innocents," is that the amendments it sustained in committee were of too sweeping a nature, so far as placing the *entire* appointment of officials in the hands of the Local Boards of Governors, which would not have been a step in the right direction in the sister country, but exactly the reverse. The principal executive appointments in them, we have no hesitation in saying, ought to be vested solely in Government, as they are at present; otherwise the Irish Asylums would soon degenerate from the high position they have so creditably and justly reached, and ultimately become mere offshoots of the Union Workhouses. With regard to the vexed question of Chaplains, we are clearly of opinion that their appointment in Ireland should not be *compulsory*, as was, and evidently still is, aimed at by certain parties, but simply permissive; and that the Local Boards themselves, being for many reasons the best judges of the necessity or otherwise of such functionaries, should be the authority to take the initiative in their appointment. Another cause we have for not weeping excessively at the fate of "No. 2 Bill" is, that its superannuation clause did not, in our mind, go far enough, inasmuch as it gave no claim to a pension, such being made to hinge upon, firstly, the recommendation of the Inspectors; and secondly, it being a *sine qua non* that the party seeking such should be proved to be the subject of mental or bodily infirmity. Under a certain term of years, we think such a restriction as the latter would be only reasonable—indeed, indispensable. But in the case of those who had faithfully and efficiently served twenty years in the anxious and unspeakably arduous duties of unbroken attendance in the care and management of a public asylum—duties which will surely be admitted to be of a most harassing and trying nature—we hesitate not to say that they are pre-eminently entitled to claim as a right the enjoyment of their full salary thus fairly and hardly won; for we maintain, without fear of contradiction, that twenty years of unremitting labour in the daily—and nightly, too—anxieties of a lunatic asylum, are fully equal to double that number in any other department of the public service, and should be requited accordingly; the wear and tear of mind and body the individual so circumstanced has sustained, most righteously deserving this small reward for all that he has undergone during that period of time in the discharge of duties impossible to overestimate in their importance to society at large. We accordingly objected to this clause in "Bill No. 2" in our last number, seeing that it was so scant and illiberal in letter and spirit, and therefore were pleased that the Bill fell to the ground, in the full expectation that a better, to say nothing of a worse one, would be forthcoming in the next meeting of Parliament. But, sooner than we anticipated, the pensioning of the Irish Asylum Officials was again specially taken up, just before the late Session concluded, by a private member of the House; Sir Robert Ferguson, of the city of Derry, bringing in a Bill for that worthy object alone, the same proving to be *totidem verbis* the precise clause as contained in the defunct "Bill No. 2" of Government. This new Bill passed the several stages of a first and second reading, and through committee, without any amendment or objection of any kind—as it did, it should be specially observed, in its original Governmental shape, as one of the clauses in "No. 2;" but on its *third* reading, Sir Robert Ferguson moved certain amendments, which were passed as a matter of course, and which were supposed to be merely *formal*, there being no debate, explanation, or remark made on them in the House, either by him or any other member, to say nothing of sufficient notice being given to parties most interested. In fact, the whole affair was quite a surprise. This Bill, however, in its new phase as an Act (19 & 20 Vic., c. 99), proved a most miserable and discreditable affair. Instead of containing the grain of wheat in the bushel of chaff which it merely did originally, it was plundered even of that simple grain, those amendments referred to of Sir Robert's placing the conferring of pensions under the regulations of the Civil Service Pension Act (4 & 5 Wm. IV.), which, for seventeen years' service, *handsomely* allows a party to *crave*, as a matter of grace and favour, *three-twelfths* of the paltry and inadequate salary he might have enjoyed, and so on up to *fifty* years' service, when the *maximum* of eight-twelfths is all that would be granted; and not even then, unless the party had plaintively repeated "pity the sorrows of a poor old man," by proving himself to be infirm in mind or body (both of which he could do beyond fail, only that long before he would have paid the last debt of nature), and to have "discharged the duties of his situation with diligence and fidelity" all that time! We cannot trust ourselves now to express our opinion of the conduct pursued by Sir Robert Ferguson in relation to the above short and simple statement of facts. By the courtesy of Parliament, he has the title of "honourable;" nor do we mean to hint that he is not entitled to such a style of address, or that anything not perfectly honourable and straightforward was intended in the above proceedings in the "Honourable House," in which he was the *facile princeps* on this occasion; but this we will say, that the whole proceeding bears a very extraordinary aspect, and loudly calls both for explanation, and a repeal of an Act passed under such remarkable circumstances, and which is neither more nor less than a sham. And further, as we have seen elsewhere pertinently stated on this subject, we may observe, that "if either individual members assumed to be independent, or the Legislature at large, are desirous to maintain a character for plain dealing, they should beware above all things of enactments so *smuggled* through Parliament as to compel those more immediately affected to feel almost (altogether?) as if they were simply swindled."

